# Towards Sustainable Diets: Understanding the Cognitive Mechanism of Consumer Acceptance of Biofortified Foods and the Role of Nutrition Information

**DOI:** 10.3390/ijerph18031175

**Published:** 2021-01-28

**Authors:** Amar Razzaq, Yifan Tang, Ping Qing

**Affiliations:** College of Economics and Management, Huazhong Agricultural University, No. 1 Shizishan Street, Hongshan District, Wuhan 430070, China; amar.razzaq@hotmail.com (A.R.); yifan.tang@webmail.hzau.edu.cn (Y.T.)

**Keywords:** micronutrient malnutrition, biofortified foods, nutrition traits in food, nutrition information, consumer behavior, purchase intentions, ordered logistic regression, sustainable diets

## Abstract

Micronutrient malnutrition, also known as hidden hunger, continues to affect more than 2 billion people globally. Biofortification, which is a process of breeding staple crops with improved micronutrient contents such as vitamin A, iron, and zinc, offers a cost-effective and sustainable solution in reducing hidden hunger. However, the success of these foods depends on consumer acceptance. In contrast to previous studies, this research focuses on the mechanism of consumer acceptance of biofortified crops that undergo physical changes (i.e., changes in appearance) after biofortification. We use data on 473 Chinese consumers collected through online surveys to examine their purchase intentions for biofortified foods that have visible (vs. invisible) nutrition traits. Using two online surveys, we conduct two studies to reveal the mechanism and antecedents of consumer acceptance of different biofortified foods. In Study 1, we find that consumer purchase intentions vary depending upon the visibility of nutrition traits in biofortified foods. Specifically, consumers exhibit a nutrition-related food neophobia (NFN) regardless of visibility of the nutrition trait in biofortified foods; and a sensory-affective food neophobia (SFN) which is only pronounced when the nutrition trait in biofortified foods is visible. The results of the mediation analysis show that for both types of biofortified foods, NFN mediates consumers’ purchasing intentions. For foods that involve visible changes after biofortification, SFN acts as an additional mediator of consumer purchase intentions. Using ordered logistic regression analysis, we find that both food neophobias have a negative impact on consumer acceptance of biofortified foods. The results of Study 2 confirm our findings and further show that nutrition information moderates the mediation of NFN and SFN, i.e., the negative impact of NFN and SFN on purchase intentions can be reduced by providing nutrition information to consumers. The results of this study have several theoretical and practical implications and are of interest to stakeholders and marketers in the promotion of biofortified foods.

## 1. Introduction

Micronutrient malnutrition, also known as hidden hunger, continues to affect more than 2 billion people globally [1]. This problem is widespread in Asia and Africa, where people rely heavily on staple foods resulting in low dietary diversity and micronutrient malnutrition. Biofortification—a process of breeding staple crops with improved micronutrient contents such as vitamin A, iron, and zinc—has been proposed as the potential solution to combat hidden hunger, bridge the gap between agriculture and nutrition, and build sustainable food systems in low and middle-income countries [2,3,4]. However, the success of biofortified crops depends on consumer acceptance.

Over the past decade, researchers have shown an increased interest in consumer acceptance of country-specific biofortified crops [5,6,7,8]. The results of these studies are encouraging, suggesting that consumers prefer biofortified foods [9,10]. However, there is no conclusive evidence that if consumers will also prefer those crops for which the biofortification changes their appearance. For example, vitamin A biofortification of some crops changes their color from white/creamy to yellow/orange because of the increased beta-carotene content. Although the biofortified crops with invisible nutrition traits are generally preferred by consumers, the results are mixed for crops involving visible nutrition traits [9,10]. In addition, most studies focusing on consumer acceptance of such crops have only assessed the impact of nutritional information on consumer acceptance [6,11,12,13], and ignored the psychological mechanism that determines consumers’ acceptance of these crops.

According to some food neophobia literature, some people may be afraid of novel foods [14,15] or foods processed with a novel technology [16]. Some consumers may reject biofortified crops with visible nutritional traits based on their unfamiliar appearance. However, there is lack of research on the effects of food neophobia on consumer acceptance of biofortified foods with visible nutrition traits. In addition, whether consumers can directly reject certain biofortified foods whose appearance has changed is also a question worth considering. Thus, the present research aims to explore the mechanism of consumer acceptance of biofortified foods by extending the food neophobia framework to these foods. Specifically, we examine the differences in consumer acceptance of different biofortified food (i.e., foods with visible and invisible nutrition traits) and its mechanism in different situations. In addition, we investigate the role of nutritional information in moderating the mechanism of consumer acceptance of different biofortified foods.

Micronutrient malnutrition is a serious problem in China as 200 million Chinese continue to suffer from micronutrient deficiencies [17]. A recent study found that Chinese adults (18–59 years old) have poorer diets and their intake is much lower than the estimated average requirements for several key micronutrients [18]. Biofortified crops are rich in micronutrients and can help alleviate China’s micronutrient malnutrition. However, there is a lack of research on Chinese consumers’ acceptance of biofortified foods. Considering the severity of China’s malnutrition problem and the lack of research on the mechanism and antecedents of acceptability of biofortified foods, this study uses data on Chinese consumers to investigate their acceptance of biofortified foods. The results of this study not only make theoretical contribution to research on consumers’ acceptance of biofortified foods, but also have practical significance for the promotion of biofortified foods to reduce global micronutrient malnutrition and establish a sustainable food system.

### 1.1. Literature Background and Hypothesis Development

#### Food Neophobia and Consumer Acceptance of Novel Foods

In the domain of functional foods (FFs), prior research shows that the FFs behavior is influenced by many psychological factors, such as general perception and attitude, beliefs and values, motivation, trust/confidence, health orientation, and neophobia [19,20]. Food neophobia is defined as a tendency to avoid novel/unfamiliar foods [21]. Although it is a characteristic of humans in general, substantial individual differences exist making some people far more resistant to trying new foods. Pliner and Hobden [22] conceptualized food neophobia as a personality trait and developed the Food Neophobia Scale (FNS). Studies that examined the relationship between food types and food neophobia have generally focused on “ethnic” foods, which may represent a cultural novelty. However, in addition to the ethnic cuisine, several other types of novel foods have also been identified. Examples of these foods include functional foods, genetically modified products, nutritionally modified foods, and organic foods [23]. Based on this classification, we argue that biofortified foods also represent a type of novel food because they are nutritionally modified foods.

While the FNS has been proven successful in measuring consumer receptivity to novel foods [24,25], it is not clear if it can be used to measure different dimensions of food neophobia related to biofortified foods. Biofortified foods, particularly those involving visible nutrition traits, have two significant attributes that may affect the food decision process, i.e., modified nutrition and color change. Prior research shows that taste, sensory appeal, nutrition content/type, price, and attitudes are important factors affecting food choice [26,27,28,29,30]. Food choice also depends on the relevant importance of these attributes for individual consumers. For instance, Mai and Hoffman identified two major consumer segments based on individual preferences: Taste lovers and nutrition fact-seekers. They showed that while rating the importance of various food attributes, taste lovers primarily considered health-unrelated product attributes whereas nutrition fact-seekers put a high emphasis on health-related attributes [31]. In the domain of novel foods, McFarlane and Pliner found consumers’ attitude toward nutrition to be a significant factor that determined the effect of general nutrition information on their willingness to taste novel foods [32].

Besides the nutritional value of foods, studies have shown that food preferences are strongly affected by sensory attributes [33]. Taste is often the most critical sensory attribute that affects what consumers eat [34]. However, the effect of visual sensation should not be underestimated. Human preference for quality is dependent on visual images [35]. A considerable amount of literature shows that food color and appearance can have a halo effect that changes the subsequent flavor perception, taste quality, and palatability of food which may ultimately affect food acceptability [36]. In addition, visual cues in food may affect the levels of food neophobia and food consumption among children [37].

The preceding discussion shows that nutritional content and appearance of food are important determinants of food choice which may also affect levels of food neophobia. Since biofortified foods with visible nutrition traits involve changes in their nutritional contents and appearance, therefore, we argue that food neophobia as behavior can be of two types: Nutrition-related food neophobia (NFN) and sensory-affective food neophobia (SFN).

We propose this classification of food neophobia by borrowing Rozin and Fallon’s classification of the sources of food refusal. According to them, food rejection in humans can originate from one (or more) of the three sources: (i) Dislike of its sensory characteristics; (ii) danger, a fear of negative consequences of eating it; and (iii) disgust arising from the idea of the food’s nature or origin [38,39]. The first two of these food rejection sources seem more relevant to biofortified foods because these foods involve nutritional modification, as well as physical changes in the food. In this study, we chose two different types of biofortified foods as stimuli based on the nutrition traits present in the crop: A biofortified food with invisible nutrition traits that exactly looks like its conventional counterpart, and a biofortified food with visible nutrition trait that changes its color.

Hence, we define nutrition-related food neophobia (NFN) as consumers’ resistance to the consumption of food caused by its nutritional aspects. These consumers might fear the negative consequences of consuming nutritionally modified food. Whereas we define sensory-affective food neophobia (SFN) as consumers’ resistance to the consumption of food caused by its sensory attributes (such as color, texture, temperature, and aroma). We propose that consumers will exhibit nutrition-related or sensory-affective food neophobia depending upon their nutrition-seeking and sensory appeal-seeking goals in food consumption. Thus, we propose that consumers facing biofortified food with an invisible (but known or believed) nutrition trait may exhibit a nutrition-related food neophobia (NFN). For biofortified foods that undergo a visible change after biofortification, not only nutritional content is modified but the appearance is also changed. Therefore, we further propose that offering consumers biofortified food with a visible nutrition trait may cause not only NFN but also a sensory-affective food neophobia (SFN). Therefore, we hypothesize that:

**Hypothesis** **1** **(H1).** *Different types of nutrition traits in biofortified foods will cause different types of food neophobia in consumers*.

(a)
  **Hypothesis** **1a** **(H1a).** *Biofortified foods with invisible nutrition traits (compared to ordinary rice) will cause nutrition-related food neophobia (NFN) in consumers*.
(b)
  **Hypothesis** **1b** **(H1b).** *Biofortified foods with visible nutrition trait (compared to the ordinary rice) not only cause nutrition-related food neophobia (NFN), but also sensory-affective food neophobia (SFN) in consumers*.

A considerable amount of literature shows that food technology neophobia negatively affects functional food behavior [40,41,42]. Therefore, we anticipate that both NFN and SFN will reduce consumer acceptance of biofortified foods. Further, we also hypothesize that the presence of invisible (visible) nutrition traits in biofortified foods will increase consumers’ NFN (SFN) levels, and these increased levels of NFN and SFN will reduce consumer purchase intentions on biofortified foods. In other words, we underscore the negative effect of food neophobia by predicting a mediation of food neophobia in the relationship of different types of biofortified foods and consumer purchase intentions. We anticipate that food neophobia will explain the differences in consumer purchase intentions on different types of biofortified foods. Based on this discussion, we propose the following hypotheses:

**Hypothesis** **2** **(H2).** *The nutrition-related food neophobia (NFN) and sensory-affective food neophobia (SFN) will reduce consumers’ purchase intention on biofortified foods*.

(a)
  **Hypothesis** **2a** **(H2a).** *NFN will reduce consumers’ purchase intention on biofortified foods*.
(b)
  **Hypothesis** **2b** **(H2b).** *SFN will reduce consumers’ purchase intention on biofortified foods*.

**Hypothesis** **3** **(H3).** *Food neophobia (NFN and SFN) mediates the relationship between biofortified food-type and consumer purchase intentions*.

### 1.2. The Effect of Nutrition Information on Food Neophobia and Food Choice

Rozin argues that foods that taste good and are considered beneficial by consumers are often the most acceptable foods [43]. Previous studies have proved the usefulness of positive taste information on consumer acceptance of novel foods [32,43,44,45,46] and biofortified foods [47]. Evidence also exists that positive health information on novel foods can reduce consumers’ food neophobia and increase their willingness to try novel foods [32]. Therefore, we anticipate a similar effect in the context of biofortified foods:

**Hypothesis** **4** **(H4).** *Nutrition information about the health benefits of biofortified foods will directly moderate the relationship between types of biofortified food and consumers’ food neophobia (NFN and SFN), thereby indirectly moderating the relationship between types of biofortified food and consumers’ purchase intentions*.

**Figure 1:**
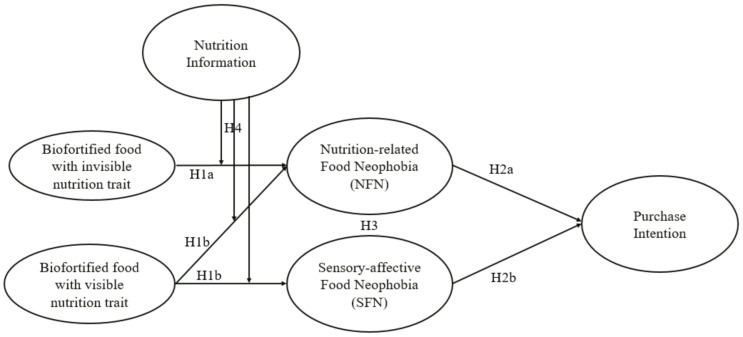
The conceptual model of the study.

In summary, we predict that exposure to biofortified foods with invisible nutrition trait will cause nutrition-related food neophobia (NFN) in consumers. In contrast, biofortified foods with visible nutrition trait will not only cause NFN but also a sensory-affective food neophobia (SFN) in consumers. The resultant food neophobia will act to reduce consumer purchase intentions (PI) for biofortified foods and explain the differences in consumers’ purchase intentions (PI). Further, we predict that providing consumers with nutritional information about the health benefits of the nutrition trait present in biofortified foods will reduce the food neophobia (NFN and SFN) associated with such foods. The conceptual model of this research is shown in Figure 1.

## 2. Research Methodology

This research used data collected from consumers through online surveys. We conducted two separate studies to test our hypotheses. An overview of these studies is provided below, while the information on study design, procedure, participants, and analytical methods is provided in relevant sections of Study 1 and Study 2.

### 2.1. Overview of Studies

We conducted two separate studies that test our hypotheses, i.e., how the presence of visible and invisible nutrition traits in biofortified foods affects consumer purchase intentions, the mediating role of food neophobia, and the moderating effect of nutrition information. Using vitamin A biofortified rice with visible and invisible nutrition trait as stimuli, Study 1 provides evidence that compared to ordinary rice, when consumers face biofortified rice with an invisible nutrition trait, they exhibit NFN. In contrast, when participants face biofortified rice with a visible nutrition trait, they exhibit not only NFN but also SFN. The results also reveal that both types of food neophobia reduce consumer purchase intentions on biofortified rice. Further, this study also confirms the mediating role of NFN and SFN in the relationship of biofortified rice-type and consumer purchase intentions.

Study 2 examines the effect of providing participants with nutrition information about the health benefits of vitamin A on food neophobia (NFN and SFN) and consumer purchase intentions. In other words, Study 2 also examines the moderated mediation of nutrition information in the relationship of biofortified rice type, food neophobia, and consumer purchase intentions.

### 2.2. Variable Measurement

NFN and SFN. Before conducting Study 1 and Study 2, we constructed scales for measuring nutrition-related (NFN) and sensory-affective food neophobia (SFN). The general food neophobia can be measured on the Food Neophobia Scale developed by Pliner and Hobden [22]. However, as we argued before that consumers will experience different types of biofortified food-related neophobia depending upon their food consumption goals, which can be of two types namely the NFN and SFN. Therefore, we first describe the NFN and SFN scales briefly.

**Figure 2:**
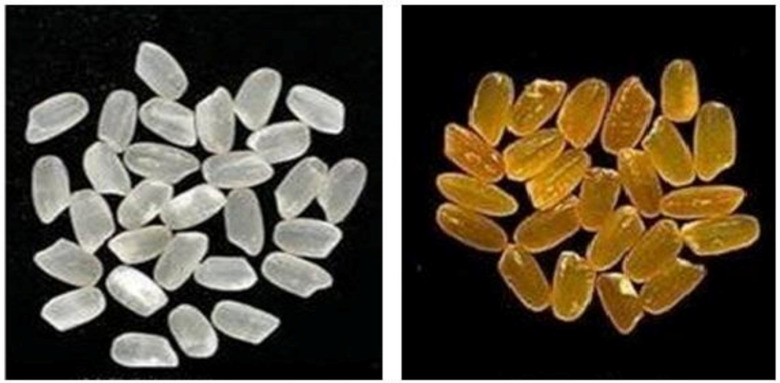
The white and yellow rice grains shown to participants indicating the presence of invisible and visible nutritional traits due to biofortification. For the control group (ordinary rice), the picture of white rice grain was shown to the participants in Study 1 and 2.

The pretest was conducted in three stages. In the first stage, undergraduate students (N = 67, average age 20.1 years, 53% females) recruited from a major university of Wuhan were invited to participate in a focus group. For discussion, we chose rice as an example biofortified product and showed them pictures of two different types of vitamin A biofortified rice (henceforth VA rice) containing invisible and visible nutrition trait (Figure 2). There were several reasons for choosing rice. First, almost all the participants reported that rice was their staple food, and they ate rice at least twice a day. Therefore, the choice of rice as the target product ensured that participants in subsequent studies took the subject of the study seriously as it was the food they could recognize and interact with it every day. Second, we needed food that was not only a staple food but also undergoes visible changes after biofortification. The vitamin A biofortification of rice changes the natural color of rice. In the focus group, participants were told to assume that biofortification could produce both types of VA rice, i.e., with visible and invisible nutrition traits. When the VA enrichment is visible, it turns the rice color from white to yellow. However, for the invisible nutrition trait, VA enrichment would not change the rice color.

Next, we described the construct of nutrition-related food neophobia (NFN) and sensory-affective (SFN) scale to the participants informally. We quoted examples of the existing Food Neophobia Scale (FNS) and informed respondents that the proposed NFN and SFN scales would be used to measure the levels of a person’s liking/disliking of biofortified foods containing invisible and visible nutrition traits, respectively. After that, we asked participants to imagine a continuum along which individuals could place themselves according to their willingness to try novel foods such as VA rice.

Participants gave about 15 statements for each of the NFN and SFN scales. The statements included positively and negatively worded statements about the consumption of biofortified rice with invisible nutrition trait (items were indicative of nutrition-related food neophobia), and visible nutrition trait (items were indicative of sensory-affective food neophobia). We supplemented the participants’ statements with 15 additional items for each scale derived from a literature search of past studies involving novel foods, food produced through new technologies, and functional foods.

In the second stage, the is resulting set of 30 items for each of the NFN and SFN scales were administered to 82 other participants (average age 20.6 years, 48.4% females) recruited from a major university of Wuhan who were shown pictures of two rice types and then rated their agreement to each of the previously identified items on a 5-point Likert scale (1 = strongly disagree, 5 = strongly agree). Preliminary analysis (ANOVA) of participants’ response scores to the proposed NFN and SFN items indicated no significant gender or age differences. We reversed the scoring of negatively worded items and calculated the total item-scores for each of the NFN and SFN scales. The uncorrected item-whole correlations were computed. In addition, the face validity of items was checked by the evaluation of items two research assistants who rated their agreements on a 5-point Likert scale (1 = strongly disagree, 5 = strongly agree). The items with low item-whole correlations and low average face validity scores were excluded, leaving 13 items for the NFN scale, and 12 items for SFN scales.

In the third stage, the resulting two sets of items from the second stage were further presented to two samples of subjects (N = 46 and 48) who were instructed to rank their agreement to each item on a 5-point Likert scale. We computed uncorrected item-whole correlations for each sample. Upon examination of the correlations of NFN and SFN items for the larger of the samples, we selected 5 items with the highest correlations whereas the average item-whole correlation for the selected set of items was 0.74 and 0.72, respectively. Next, we examined the correlations for the smaller sample and found that the same five-items set selected for each of NFN and SFN had the highest item-whole correlations in the smaller sample. These items became the NFN and SFN scales. The Cronbach’s alpha for both the scales was 0.75, which indicates higher internal consistency. In addition, the inter-rater reliability of the scales was evaluated by five researchers from different disciplines, who independently scored the scale items to indicate whether a particular item belongs to NFN or SFN scale. The intraclass correlation coefficient of 0.959 (95% CI: 0.898–0.988) was found, indicating higher inter-rater reliability.

The NFN and SFN scales used in this research are expected to negatively correlate with willingness to try the rice presented to participants, i.e., the lower scores are likely to correlate with higher willingness to try the specific rice type, while the higher scores represent a lower willingness to try that type of rice. The inspection of the frequency distribution for both NFN and SFN scores appeared to be unimodal and normal. The items used in NFN and SFN scales are displayed in Table 1.

Purchase Intentions. Consumers’ purchase intention was measured with a single item adapted from Chen [48]: “I would buy this rice with the coming day”, on a five-point Likert scale (1 = “not at all” and 5 = “absolutely”).

### 2.3. Analytical Methods

The first hypothesis, i.e., the impact of nutrition trait visibility on food neophobia was tested by analyzing the levels and significance of NFN and SFN scores across different groups of participants. The t-test was used to analyze these results. The mediation of NFN and SFN in the relationship of food type and purchase intentions (H2) was tested using mediation analysis proposed by Hayes (model 4) [49]. In addition, the moderated mediation of nutritional information (H4) was tested using moderated-mediation analysis proposed by Hayes (model 7) [50].

### 2.4. The Ordered Logistic Regression Model

The effect of food neophobia (NFN and SFN) on consumers’ purchase intentions was analyzed using ordinal logistic regression. Since the dependent variable (purchase intentions) is measured on a single item 5-point Likert scale, therefore an ordered logistic regression was deemed appropriate [51,52]. In addition to NFN and SFN, socio-demographic variables such as participants’ age, education, gender, income, and marital status were also included in the model. The cumulative probability expression of the independent variables in the ordered logistic regression model is as follows:

(1) $$P\;(Y\leq j\;|\;\underline{x}\;)=\;F_{j}\left(\underline{x}\right)\;=\pi_{1}\;\left(\underline{x}\right)\;+\cdots +\;\pi_{j}\;\left(\underline{x}\right)$$

where $\pi_{j}\;\left(\underline{x}\right)$ is the response probability of the j indicator of the explanatory variable *X*. The cumulative logits expression for each indicator j is as follows:

(2) $$L_{j}\left(\underline{x}\right)\;=\ln\;\left[\frac{F_{j}\left(\underline{x}\;\right)}{1-F_{j}\left(\underline{x}\;\right)}\right],\;j=1,\;2,\;3,\;\ldots \;\left(c-1\right).$$

All accumulated logs can be expressed as follows:

(3) $${\dot{L}}_{j}\left(\underline{x}\right)\;=\;{\dot{\propto }}_{j}+\;{\dot{\beta }}_{j}\underline{\prime x}$$

where $\propto_{j}$ is the intercept for each cumulative logit, and it increases with an increase in *j*. The expression $P\;(Y\leq j\;|\;\underline{x}\;)$ will also increase as the *j* increase, keeping the value of *x* constant. The ${\dot{\propto }}_{j}\;\mathrm{and}\;{\dot{\beta }}_{j}\prime$ in Equation (3) are the maximum likelihood estimates. The above estimates represent the cumulative logits of each indicator if other explanatory variables have no effect on $L_{j}\left(\underline{x}\right)$. ${\dot{\beta }}_{j}\prime$ is a set of regression coefficients corresponding to $\underline{x}$, which represent the cumulative logits of each indicator j.

The next step is to estimate the value of $P\;(Y\leq j\;|\;\underline{x}\;)$ which can be worked out by the following logit cumulative function:

(4) $$P\;(Y\leq j\;|\;\underline{x}\;)=\left[\frac{1}{1+\exp\;(-{\dot{\propto }}_{j}-{\dot{\beta }}_{j}\underline{\prime x}\;)}\right].$$

The result of which is obtained as shown below:

(5) $$P\;(Y\leq j\;|\;\underline{x}\;)=\left[\frac{1}{1+\exp\;(-{\dot{L}}_{j}\left(\underline{x}\;\right)\;)}\right].$$

The marginal effects were also calculated to further analyze the changes in purchase intentions. The marginal effects were calculated with the following formula:

(6) $$\left[\frac{\partial P\;Y\;\leq j\;|\;\underline{x}}{\partial x}\right]=\;\left[\frac{\exp\;(-{\dot{L}}_{j}(\underline{x}\;))\times {\dot{L}}_{j}\left(\underline{x}\;\right)}{{\left(1+\exp\;(-{\dot{L}}_{j}(\underline{x})\;(\underline{x}\;)\right)}^{2})}\right].$$

Next, we describe the procedures and results of the two studies which we conducted to test our hypotheses.

## 3. Study 1

The main purpose of Study 1 is to test the effect of rice types on food neophobia. Further, we also investigate the influence of food neophobia on purchase intentions and the mediating role of food neophobia in the relationship between rice type and purchase intentions. We predict that compared to ordinary rice, the presence of visible (vs. invisible) nutrition trait in VA rice would cause both SFN and NFN (vs. just NFN) food neophobia in consumers (Hypothesis 1). In turn, SFN (NFN), caused by the presence of visible (invisible) nutrition trait in rice would reduce consumer purchase intentions (Hypothesis 2), and NFN and SFN will mediate this effect (Hypothesis 3).

### 3.1. Design and Procedure

One hundred and sixty-one Sojump (The Sojump is one of the most popular online survey platforms in China) participants (56.6% female; M_age_ = 29.65, SD_age_ = 7.15) were recruited and randomly assigned to one of three conditions: VA rice with invisible nutrition trait vs. VA rice with visible nutrition trait vs. ordinary rice.

We used pictures of white rice and yellow rice to manipulate the nutrition trait. In particular, participants in the invisible trait condition saw a picture of white VA rice, whereas participants in the visible nutrition trait condition saw a picture of yellow grains of VA rice (Figure 2). Participants were first asked to look carefully at the picture and read the description. The description of each VA rice stated that the product in question was developed through a method referred to as biofortification, which involves the natural breeding of crops to increase their nutritional content. We informed participants in the invisible nutrition trait group that the only change in the VA rice was VA enrichment. The participants in the visible nutrition trait group were told that the color of this VA rice was yellow due to higher levels of beta-carotene which provided vitamin A. Participants in both VA rice conditions were informed that the VA rice shown in the picture did not differ from ordinary rice in its other sensory properties (i.e., taste, aroma, and texture). After looking at the pictures and reading the description, the participants answered if they noticed the VA biofortification in the rice, and then completed measures of NFN, SFN, and purchase intention (PI). Finally, participants’ demographic information was collected.

The results showed that all subjects could recall the treatment, indicating that stimuli implemented in the study were effective. Notably, we did not provide health-related information on VA rice to any of the participants in Study 1. Participants in the third group, i.e., ordinary rice (control) condition just saw a picture of ordinary rice and did not receive any description of biofortification (Figure 2).

### 3.2. Results

Nutrition-related food neophobia (NFN): An independent sample t-test showed that participants’ rating on NFN in the invisible nutrition trait condition was significantly higher than the control condition (M_invisible_ = 15.14, SD = 3.85; M_control_ = 5.87, SD = 0.87; t(103) = 17.22; *p* < 0.001), indicating that invisible biofortification trait increased consumers’ NFN on rice. Also, consumer ratings on NFN in the visible nutrition trait condition were significantly higher than the control group (M_visible_ = 14.66, SD = 4.03; M_control_ = 5.87, SD = 0.87; t(108) = 15.68; *p* < 0.001) indicating that biofortification also increased consumers’ NFN on rice when nutrition trait is visible. However, we did not observe a significant difference in NFN score of invisible and visible nutrition trait groups (M_invisible_ = 15.14, SD = 3.85; M_visible_ = 14.66, SD = 4.03; t(105) = 0.62; *p* = 0.53). These results show that NFN is caused under both types of biofortification conditions.

**Figure 3:**
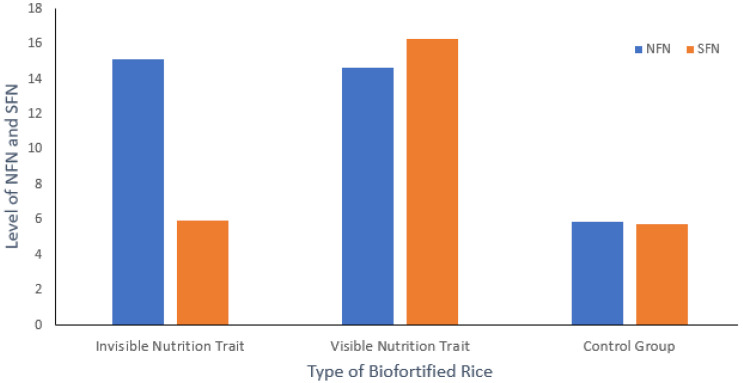
Participants’ ratings on NFN and SFN on different rice types.

Sensory-affective food neophobia (SFN). An independent sample t-test showed that participants’ ratings on SFN in visible nutrition trait condition were significantly higher than invisible trait condition (M_visible_ = 16.30, SD = 4.41; M_invisible_ = 5.96, SD = 0.96; t(105) = −16.41; *p* < 0.001), indicating that visible biofortification trait in VA rice increased consumers’ SFN on rice. Further, we find that participants’ rating on SFN in the visible nutrition trait group was significantly higher than the control group (M_visible_ = 16.30, SD = 4.41; M_control_ = 5.72, SD = 0.81; t(108) = 15.68; *p* < 0.001). However, we did not observe a significant difference in SFN scores of participants in the invisible trait and control condition (M_invisible_ = 5.96, SD = 0.96; M_control_ = 5.72, SD = 0.81; t(103) = 1.38; *p* = 0.17). These results show that SFN is only caused when the nutrition trait in VA rice is visible. Based on these results, we conclude that the invisible nutrition trait in VA rice only causes NFN, and the visible trait in VA rice causes not only cause NFN but also SFN (Figure 3). These findings thus support H1.

Impact of food neophobia on purchase intentions. To test the impact of food neophobia (the NFN and SFN) on consumer WTP, we used an ordered logistic regression model with purchase intentions as the dependent variable, and NFN and SFN as independent variables. The ordered logistic regression model was used because the dependent variable PI is ordinal and is measured on a five-point Likert scale. Several control variables such as age, gender, marital status, education, and income were also included in the regression analysis. Regression results are provided in Table 2. The results revealed a significant negative effect of NFN and SFN on consumer purchase intentions. In particular, a unit increase in NFN score would result in a 0.28 unit decrease in ordered logit odds of being in a higher VA rice purchase intention category. Similarly, a one-unit increase in SFN would result in a 0.12 unit decrease in ordered-logit odds of being in a higher VA rice purchase intention category. The effect of other variables was not significant. Marginal effects of the regression model were also estimated. These results clearly suggest that NFN and SFN are inversely related to consumer purchase intentions. In other words, consumers’ intention to purchase VA rice will decrease with the increased levels of NFN and SFN. These results support the hypothesis H2, i.e., food neophobia (the NFN and SFN) reduce consumer purchase intentions for biofortified rice.

Mediation Analysis. To investigate the mediating role of food neophobia (NFN and SFN) in the relationship of rice type and purchase intentions, we conducted three mediation analyses following Hayes [50]. The results are as follows:

First, we used rice type (invisible nutrition trait = 1, control group = 0) as an independent variable, NFN and SFN as mediators, and purchase intentions as the dependent variable. A 10,000 resample bootstrap analysis revealed that the indirect effect of NFN was significant (β = 0.956, SE = 0.165, 95% CI is 0.625 to 1.281). This result confirmed the mediating role of NFN. However, the indirect effect of SFN is not significant (β = −0.10, SE = 0.018, 95%CI is −0.054 to 0.021), indicating that only NFN is evoked when nutrition trait is invisible. This result supports the hypothesis H3a.

Second, we used rice type (visible nutrition trait = 1, control group = 0) as an independent variable, NFN and SFN as mediators, and purchase intentions as the dependent variable. A 10,000 resample bootstrap analysis revealed that the indirect effect of NFN was significant (β = 1.255, SE = 0.322, 95% CI is 0.630 to 1.892). In addition, we find that the indirect effect of SFN is also significant under this condition (β = 0.819, SE = 0.324, 95% CI is 0.180 to 0.146). These results indicate that when nutrition trait in VA rice is visible, both NFN and SFN mediate the relationship of VA rice type and purchase intentions. These results support H3b.

Third, we used rice type (invisible nutrition trait = 0, visible nutrition trait = 1) as an independent variable, NFN and SFN as mediators, and purchase intentions as the dependent variable. A 10,000 resample bootstrap analysis revealed that the indirect effect of SFN was significant (β = −0.630, SE = −0.323, 95% CI is −1.278 to −0.028). However, the indirect effect of NFN is not significant (β = 0.080, SE = 0.129, 95% CI is −0.186 to 0.325). These results indicate that SFN is the main mechanism for affecting purchase intentions because NFN is evoked under both types of VA rice, but SFN is evoked only when nutrition trait is visible.

## 4. Study 2

The main purpose of Study 2 is to investigate the moderating effect of nutrition information. Here, in addition to manipulating rice types, we also manipulate the nutrition information regarding the health benefits of VA. We predict that nutrition information will directly affect the relationship of rice type and food neophobia by reducing NFN and SFN, while it will indirectly improve purchase intentions through its impact on food neophobia. By demonstrating the moderating effect of nutrition information, this study provides further support to our earlier results that food neophobia plays a mediating role in the relationship of rice type and consumer purchase intentions. Further, it also provides insight into way to reduce consumers’ NFN and SFN through nutrition information.

### 4.1. Design and Procedure

Three hundred and twelve Sojump participants (52.6% female; Mage = 31.65, SDage = 7.15) were recruited and randomly assigned to one of 6 conditions in a 3 (Rice type: Visible rice vs. invisible rice vs. ordinary rice) × 2 (Nutrition information: Yes vs. No) between-subject design.

As in Study 1, the participants in the invisible and visible nutrition trait group were first shown the picture and description of VA rice, while those in the ordinary rice group were shown the picture of ordinary rice. Participants were first asked to look carefully at the picture and read the description. The description of each VA rice stated that the product in question was developed through a method referred to as biofortification, which involves natural breeding of crops to increase their nutritional content and did not differ from ordinary rice in its other sensory properties (i.e., taste, aroma, and texture). Further, we informed participants in the invisible nutrition trait group that the only change in the VA rice was VA enrichment. In addition to this description, participants in the nutrition information condition read a script containing the information on the health benefits of the vitamin in diets, as well as, the consequences of VA deficiency, particularly for mothers and young children. After reading the relevant description and looking at the pictures, participants completed the measure of NFN, SFN, and purchase intentions. Finally, participants’ demographic information was collected.

### 4.2. Results

We predicted that nutrition information will directly affect the relationship of rice type and food neophobia by reducing both food neophobia, while it will indirectly improve purchase intentions through its impact on food neophobia. We confirmed this by running three separate moderated-mediation analyses for the three comparison groups. In each analysis, we see the mediating role of NFN and SFN under different information conditions. We also test whether information moderates this mediation. The results are described below.

First, we performed a moderated mediation analysis following Hayes [49] with rice type (invisible nutrition trait = 1, ordinary rice = 0) as an independent variable, nutrition information (yes = 1, no = 0) as moderator, NFN, and SFN as mediators, and purchase intentions as the dependent variable. A 10,000 resample bootstrap analysis revealed that the indirect effect of NFN on purchase intentions is significant for participants who did not receive nutrition information (β = 1.189, SE = 0.071, 95% CI is 1.051 to 1.332). However, this effect disappears when nutrition information is provided to the participants (β = 0.020, SE = 0.010, 95% CI is −0.264 to 0.208). The moderated meditation model of NFN is significant (β = 0.148, SE = 0.084, 95% CI is −0.314 to −0.016). However, the indirect effect of SFN was not significant in nutrition information (β = 0.001, SE = 0.008, 95% CI is −0.014 to 0.016) and no nutrition information (β = 0.001, SE = 0.007, 95% CI is −0.013 to 0.019) conditions. The moderated mediation model of SFN was also not significant (β = 0.001, SE = 0.011, 95% CI is −0.025 to 0.021). These results indicate that for VA rice with invisible nutrition trait, the moderated mediation of nutrition information in the relationship of rice type and food neophobia will work through NFN only. Further, this effect will lead to improved purchase intentions toward such type of rice.

Second, we performed a moderated mediation analysis following Hayes [49] with rice type (visible nutrition trait = 1, ordinary rice = 0) as an independent variable, nutrition information (yes = 1, no = 0) as moderator, NFN, and SFN as mediators, and purchase intentions as the dependent variable. A 10,000 resample bootstrap analysis revealed that the indirect effect of NFN on purchase intentions is significant when nutrition information is not provided to the participants (β = 0.984, SE = 0.161, 95% CI is 0.048 to 0.410). However, this effect is disappeared (β = 0.179, SE = 0.129, 95% CI is −0.052 to 0.453) when nutrition information provided to the participants. The moderated meditation of the NFN model is confirmed by its significance (β = −0.274, SE = 0.134, 95% CI is 0.069 to 0.216). In addition, we find that the indirect effect of SFN was significant for participants under no nutrition information condition (β = 1.232, SE = 0.155, 95% CI is 0.934 to 1.546). However, the indirect effect of SFN disappears when information is provided to the participants (β = 0.063, SE = 0.018, 95% CI is −1.251 to 0.214). The moderated mediation of the SFN model is confirmed by its significance (β = −0.370, SE = 0.187, 95% CI is −0.732 to −0.004). These results indicate that for VA rice with visible nutrition trait, nutrition information to participants moderates the relationship of rice type and food neophobia through both the NFN and SFN. In other words, the effect of NFN and SFN disappears when participants receive nutrition information related to VA rice. Further, the results confirm that the moderated mediation effect of nutrition information will lead to improved purchase intentions toward such type of rice.

Third, we performed a moderated mediation analysis following Hayes [49] with rice type (invisible nutrition trait = 1, visible nutrition trait = 0) as an independent variable, nutrition information (yes = 1, no = 0) as moderator, NFN, and SFN as mediators, and purchase intentions as the dependent variable. A 10,000 resample bootstrap analysis revealed that the indirect effect of SFN on purchase intentions was significant when participants had no nutrition information (β = −0.873, SE = 0.136, 95% CI is −1.147 to −0.616), and this effect disappeared after providing nutrition information to the participants (β = 0.062, SE = 0.081, 95% CI is −0.036 to 0.143). Also, the moderated mediation of SFN was significant (β = 0.255, SE = 0.133, 95% CI is 0.023 to 0.529). However, in this case, the indirect effect of NFN is not significant whether nutrition information is provided (β = −0.028, SE = 0.112, 95% CI is −0.254 to 0.183) or not provided (β = −0.019, SE = 0.101, 95% CI is −0.214 to 0.182). In addition, the moderated mediation is also not significant for NFN (β = −0.009, SE = 0.151, 95% CI is −0.325 to 0.267). These results confirm our earlier result that NFN is evoked for both types of VA rice, while SFN is only evoked for VA rice with the visible trait. And in this case, the nutrition information moderates the relationship of rice type and food neophobia through SFN only. The NFN becomes irrelevant because the nutrition content has been modified in both types of rice.

## 5. Discussion and Conclusions

Biofortified crops are being promoted as the potential solution to reduce hidden hunger [2] because these crops offer a cost-effective and sustainable solution to the problem of micronutrient malnutrition. However, the consumer acceptance of these crops might be challenging because of several reasons. Stakeholders are continually looking for ways to increase the acceptance of these crops by large populations. Available evidence from many countries suggests that consumers prefer biofortified crops [9]. However, more research is needed to understand the broad range of factors that may reduce consumer acceptance and the ways to promote biofortified crops. Specifically, the mechanism of consumer acceptance of these foods has not been well studied. Since plenty of evidence exists that consumer may refuse foods that look unfamiliar to them, therefore, it is especially important to understand the mechanism of consumer acceptance of biofortified foods which undergo change in appearance after biofortification.

This research fulfills this gap in the literature by focusing on uncovering the mechanism of consumer acceptance of biofortified foods that have visible (invisible) nutrition traits. Building on previous research on consumer acceptance of novel foods, we extend the framework of food neophobia to biofortified foods and test whether consumers’ food neophobia has different dimensions based on the visibility of nutrition trait in the food. The data used in this study came from two online surveys on Chinese consumers. Through these surveys, we compare consumer acceptance of biofortified foods with visible and invisible nutrition traits. The stimuli used in the research were pictures of vitamin A (VA) biofortified rice having visible and invisible nutrition traits, and all results were compared with ordinary rice as well. We predicted that consumers would experience different types of food neophobia depending upon the visibility of nutrition trait in the product, and these food neophobias will mediate consumer purchase intentions on such foods. We also anticipated that consumers’ food neophobia and purchase intentions will be moderated by the availability of nutrition information to consumers.

Two separate studies were conducted to tests the hypotheses. The findings of the two studies demonstrated that initially, consumers had lower preferences for both types of VA rice, compared to ordinary rice. However, the mechanism of consumer refusal of these two biofortified rice is different. When facing biofortified rice with the invisible trait, the consumer may feel nutrition-related food neophobia (NFN), which in turn leads to lower purchase intention. For VA rice with visible nutrition trait, consumers may exhibit not only nutrition-related food neophobia (NFN) but also a sensory food neophobia (SFN), which will further reduce consumer acceptance.

However, the effects of NFN and SFN can be addressed. The result of study 2 revealed that nutrition information may be an effective intervention, namely a salient moderator in the relationship of VA rice type and purchase intention. When consumers were provided with nutrition information about the health benefit of nutrition traits, the food neophobia caused by consumer fear of biofortification was reduced, and consumer acceptance of biofortified rice was increased. However, when we did not provide nutrition information, nutrition-related food neophobia or both nutrition-related food neophobia and sensory food neophobia were still the reason for consumers’ refusal of VA rice with invisible and visible nutrition traits, respectively.

### 5.1. Theoretical Contributions

Past studies have shown that food neophobia can reduce consumer willingness to try functional foods, as well as other novel foods [40,53,54]. However, the psychological mechanism of consumer acceptance of staple foods such as biofortified foods is not well understood. In this study, firstly we explored the consumer preference or purchase intention for biofortified food with invisible and visible nutrition traits, finding that consumers may refuse both biofortified food to some extent. In doing so, we extended the food neophobia framework to biofortified foods. Different from the previous studies, we find two unique dimensions of food neophobia, i.e., nutrition-related food neophobia and sensory-affective food neophobia according to consumer perception of different biofortified food and revealed the underlying mechanism of consumer refusal of different types of biofortified foods. Lastly, we found that providing nutrition information can increase the consumer purchase intention on both biofortified foods through lowering food neophobia, which further confirms the mediation effect of food neophobia and the moderating effect of nutrition information.

### 5.2. Managerial Implications

The findings of this study contribute to the understanding of the nature of biofortified foods-related food neophobia, the effects of visibility of a nutrition trait on food neophobia, and the role of nutrition information in reducing food neophobia. These results could help marketers in their efforts to increase consumer acceptance of such biofortified foods which involve visible changes in biofortified foods. With the increasing per-capita income, Chinese consumers are paying more and more attention to the consumption of food that is not only healthy but also satisfies their sensory needs. While the presence of a visible nutrition trait in biofortified foods may cause specific food neophobic behavior; nutrition information may reduce this food neophobia. Our findings suggest that instantaneous information on the health benefits of micronutrients (e.g., vitamin A) may be enough to increase consumer acceptance of biofortified foods with invisible and visible nutrition traits. However, additional efforts may be required to address the consumers’ concerns about physical changes in the food. Our findings also underscore the need for using different marketing strategies for different types of biofortified foods.

### 5.3 Limitations

There are a few limitations to our study. First, we conducted the two studies using online surveys. However, providing actual biofortified foods to consumers and recording their actual food neophobic behavior may reduce the likelihood of hypothetical bias. Second, the data was collected through randomly selected participants from the consumer pool available to the survey company. Since most Chinese consumers eat rice every day, no conditions were applied to the selection of respondents. Because the smartphone usage rate among young consumers is higher than that of the elderly, this selection procedure may bias the distribution of participants towards the younger age group. This is indicated by the average age of participants which is 29.65 years in Study 1 and 31.65 years in Study 2. Using survey design can overcome this limitation. Third, the NFN and SFN scales developed in this study correspond to the types of biofortified rice considered in this research, and further research is needed to make these scales more universal. Other avenues for future research include exploring the effects studied in this research by considering consumer heterogeneity, location, culture, and other types of biofortified foods.

## 6. Conclusions

This research focused on uncovering the cognitive mechanism of consumer acceptance of biofortified foods that have visible (invisible) nutrition traits. Building on previous research on consumer acceptance of novel foods, we extended the framework of food neophobia to biofortified foods and tested whether consumers’ food neophobia has different dimensions based on the visibility of nutrition trait in the food. Using online surveys, two separate studies were conducted to tests the hypotheses. The findings of the two studies demonstrated that initially, consumers had lower preferences for both types of VA rice, compared to ordinary rice. However, the mechanism of consumer refusal of these two biofortified rice is different. When facing biofortified rice with the invisible trait, the consumer may feel nutrition-related food neophobia (NFN), which in turn leads to lower purchase intention. For VA rice with visible nutrition trait, consumers may exhibit not only nutrition-related food neophobia (NFN) but also a sensory food neophobia (SFN), which will further reduce consumer acceptance. However, the effects of NFN and SFN can be addressed by providing nutrition information to consumers. When consumers were provided with nutrition information about the health benefit of nutrition traits, the food neophobia caused by consumer fear of biofortification was reduced, and consumer acceptance of biofortified rice was increased. These results indicate that providing consumers with nutritional information is an effective strategy to promote biofortified foods with visible or invisible nutritional traits.

## Figures and Tables

**Table 1:** Items used in nutrition-relation food neophobia (NFN) and sensory-affective food neophobia (SFN) scales.

**Nutrition-Related Food Neophobia (NFN) Scale**
I am afraid that this rice will harm my health.
I will try this rice only after I have made sure it is safe for consumption.
I am not willing to try this rice because I think ordinary rice has all the micronutrients I need.
I am willing to try this rice even if that makes me feel uncomfortable. [R]
I am not willing to try this rice because I fear that more than the naturally occurring amount of micronutrient in rice is not suitable for my body.
**Sensory-Affective Food Neophobia (SFN) Scale**
I will not try this rice because it does not look tasty.
This rice looks different from ordinary rice so I will not try it.
I am willing to try this rice even if its color is different from ordinary rice. [R]
I do not care about the color of rice as long as its taste does not change. [R]
I am not willing to try this rice because its appearance is not appealing.

[R] indicates reverse score items.

**Table 2:** Results of the ordered logit regression on factors influencing purchase intentions of biofortified rice.

Explanatory Variables	Ordered-Logit of PI		Marginal Effects for Various Outcomes of PI				
	β/Se	Odds Ratio	Prob (y_i_ = 1)	Prob (y_i_ = 2)	Prob (y_i_ = 3)	Prob (y_i_ = 4)	Prob (y_i_ = 5)
Age (years)	−0.001 (0.0215)	0.999	0.0001	0.0000	0.0000	0.0000	0.000
Education (years of schooling)	−0.121 (0.3188)	0.886	0.0125	0.0040	0.0002	−0.0035	−0.013
Income (US$)	0.000 (0.0000)	1.000	0.0000	0.0000	0.0000	0.0000	0.000
Gender (male = 0, female = 1)	0.323 (0.3132)	1.381	−0.0334	−0.0106	−0.0006	0.0096	0.035
married (0 = single, 1 = married)	−0.073 (0.3582)	0.929	0.0075	0.0025	0.0001	−0.0021	−0.008
NFN	−0.282 *** (0.0379)	0.754	0.0291	0.0094	0.0005	−0.0083	−0.031
SFN	−0.125 *** (0.0310)	0.882	0.0129	0.0042	0.0002	−0.0037	−0.014
Threshold Points							
/cut1	−7.284 *** (1.390)						
/cut2	−5.694 *** (1.340)						
/cut3	−4.666 *** (1.312)						
/cut4	−3.336 * (1.270)						
Model Fitness							
Pseudo R^2^	0.383						
Log Likelihood	195.509						
Log Likelihood χ^2^	113.700						
Prob > χ^2^	0.000						

Notes: *** *p* < 0.001, * *p* < 0.05; PI = purchase intentions.

## Data Availability

The data are not publicly available to protect the confidentiality of the participants.
